# MMP-7 and fcDNA Serum Levels in Early NSCLC and Idiopathic Interstitial Pneumonia: Preliminary Study

**DOI:** 10.3390/ijms141224097

**Published:** 2013-12-11

**Authors:** Paola Ulivi, Gian Luca Casoni, Giovanni Foschi, Emanuela Scarpi, Sara Tomassetti, Micaela Romagnoli, Claudia Ravaglia, Marta Mengozzi, Wainer Zoli, Venerino Poletti

**Affiliations:** 1 Biosciences Laboratory, Istituto Scientifico Romagnolo per lo Studio e la Cura dei Tumori (IRST) IRCCS, Meldola 47014, Italy; E-Mails: giova.foschi@email.it (G.F.); w.zoli@irst.emr.it (W.Z.); 2 Pulmonology, Department of Thoracic Diseases, Morgagni-Pierantoni Hospital, Forlì 47121, Italy; E-Mails: casoni1970@libero.it (G.L.C.); s.tomassetti@ausl.fo.it (S.T.); m.romagnoli@ausl.fo.it (M.R.); claudiaravaglia@alice.it (C.R.); venerino.poletti@gmail.it (V.P.); 3 Unit of Biostatistics and Clinical Trials, IRST IRCCS, Meldola 47014, Italy; E-Mail: e.scarpi@irst.emr.it; 4 Thoracic Surgery, Department of Thoracic Diseases, Morgagni-Pierantoni Hospital, Forlì 47121, Italy; E-Mail: m.mengozzi@ausl.fo.it

**Keywords:** MMP-7, fcDNA, IPF, NSIP, NSCLC, serum, IIPs

## Abstract

A non-invasive test to facilitate the diagnosis of non-small cell lung cancer (NSCLC) and idiopathic pulmonary fibrosis (IPF) is still not available and represents an important goal. Forty-eight patients with stage I NSCLC, 45 with IPF, 30 with other idiopathic interstitial pneumonias (IIPs) including idiopathic non-specific interstitial pneumonia (NSIP) and chronic hypersensitivity pneumonitis (HP), 35 with diffuse non-malignant disease and 30 healthy donors were enrolled onto the study. Free circulating (fc)DNA and MMP-7 levels were evaluated by Real Time PCR and ELISA, respectively. Median fcDNA levels were similar in NSCLC (127 ng/mL, range 23.6–345 ng/mL) and IPF (106 ng/mL, range 22–224 ng/mL) patients, and significantly lower in IIPs patients, in individuals with other diseases and in healthy donors (*p* < 0.05). Conversely, median MMP-7 values were significantly higher in IPF patients (9.10 ng/mL, range 3.88–19.72 ng/mL) than in those with NSCLC (6.31 ng/mL, range 3.38–16.36 ng/mL; *p* < 0.0001), NSIP (6.50 ng/mL, range 1.50–22.47 ng/mL; *p* = 0.007), other diseases (5.41 ng/mL, range 1.78–15.91, *p* < 0.0001) or healthy donors (4.35 ng/mL, range 2.45–7.23; *p* < 0.0001). Serum MMP-7 levels seem to be capable of distinguishing IPF patients from those with any other lung disease. fcDNA levels were similar in NSCLC and IPF patients, confirming its potential role as a biomarker, albeit non-specific, for the differential diagnosis of NSCLC.

## 1. Introduction

Non-small cell lung cancer (NSCLC) and idiopathic pulmonary fibrosis (IPF) represent two of the most serious lung diseases. The fibrotic process that characterizes IPF is commonly considered the result of a recurrent injury to the alveolar epithelium followed by uncontrolled proliferation of bronchiolar epithelium and fibroblasts [1,2]. The new pathogenetic paradigm of IPF suggests that it is a complex process characterized by abnormal pneumocyte apoptosis and profound derangement of alveolar renewal more similar to malignant lung disease [3–5]. Indeed, epigenetic and genetic abnormalities, altered cell-to-cell communications, uncontrolled proliferation and abnormal activation of specific signal transduction pathways are biological hallmarks that characterize the pathogenesis of IPF and cancer [4,6].

A differential diagnosis between IPF and other fibrosing interstitial lung diseases is very important because of differing treatment approaches and prognosis for the two types of disease. We previously demonstrated that free circulating DNA (fcDNA) levels are higher in IPF patients than in those with other interstitial lung diseases [7]. Conversely, we and other authors have also shown that fcDNA levels are significantly higher in NSCLC patients than in healthy donors or individuals with non-neoplastic disease [8–10]. Although several hypotheses have been formulated about the nature of fcDNA in cancer, the mechanisms responsible for high fcDNA levels in IPF remain to be clarified.

Circulating matrix metalloproteases 1 and 7, fibrocytes, Krebs von den Lungen (KL)-6 and surfactant proteins (SP) A and D, CCL18, (known also as pulmonary activation-related chemokine or PARC), interleukin 8 (IL-8) and monocyte chemotactic protein-1 (CCL2 or MCP-1) have been proposed as potential markers for IPF diagnosis, stimulating further research into this area [11–13]. In particular, matrix metalloproteinases (MMPs), have been investigated as potential tools to distinguish IPF from the other pulmonary diseases [11]. MMPs are endopeptidases that play an important role in the process of extracellular matrix (ECM) and basal membrane degradation and have been linked to a number of diseases [11,14]. MMP-7 (matrilysin) cleaves collagen IV, elastin, intactin, fibronectin, gelatin, laminin and tenascin, and is known to be involved in several lung malignancies and non-malignancies [14], including IPF [15] and NSCLC [14]. IPF is characterized by diffuse interstitial inflammation, fibroblast proliferation and accelerated remodeling of extracellular matrix (ECM), all of which result in irreversible destruction of the fine architecture of the lung [3–5]. It has also been shown that serum MMP-7 levels are higher in IPF patients, including asymptomatic ones, than in individuals with other non-malignant lung diseases, indicating its potential usefulness as a marker for the early non-invasive diagnosis of IPF [11]. MMP-7 is produced mainly by glandular epithelial cells and macrophages in diseased tissues and is overexpressed by cancer cells in human tumors. It has been linked to the development of metastases [16] and is also considered to play a role in tumorigenesis and tumor progression [17]. We thus aimed to analyze MMP-7 and fcDNA serum levels in patients with different malignant and non-malignant lung diseases, including NSCLC, IPF and other IIPs including non-specific interstitial pneumonia (NSIP) and chronic hypersensitivity pneumonitis (HP).

## 2. Results

Patient characteristics are reported in Table 1.

### 2.1. fcDNA

**Figure 1.**
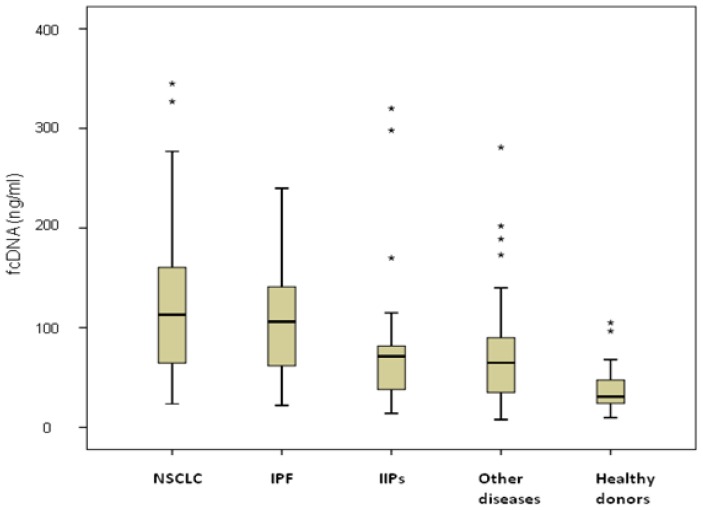
Free circulating DNA levels in healthy donors and patients with different lung diseases. The asterisks stand for outliers, *i.e*. observation points that are distant from other observations.

Similar fcDNA levels were observed in NSCLC and IPF patients (127 ng/mL, range 23.6–345 ng/mL, and 106 ng/mL, range 22–224 ng/mL, respectively), both significantly higher than those of IIPs patients (72.5 ng/mL, range 14–320, *p* = 0.012 and *p* = 0.045, respectively), individuals with other diseases (68.3 ng/mL, range 7.8–281 ng/mL, *p* = 0.049 and *p* = 0.046, respectively), and healthy donors (30.8 ng/mL, range 9.7–105 ng/mL, *p* < 0.001) (Figure 1). Among NSCLC patients, no differences were observed between those with COPD (19 patients) or without COPD (28 patients), with median values of 127 ng/mL (range 31.2–327 ng/mL) and 124.5 ng/mL (range 23.6–345 ng/mL), respectively, (*p* = 0.530). Moreover, considering all COPD patients, higher fcDNA median values were observed in those with cancer (127 ng/mL, range 31.2–327 ng/mL) than in those with COPD alone (66.6 ng/mL, range 14–281 ng/mL) (*p* = 0.06). fcDNA levels were not associated with either clinical-pathological characteristics of patients such as age, gender and smoking habits, or with clinical features of the various diseases (data not shown).

### 2.2. Serum MMP7 Levels

**Figure 2.**
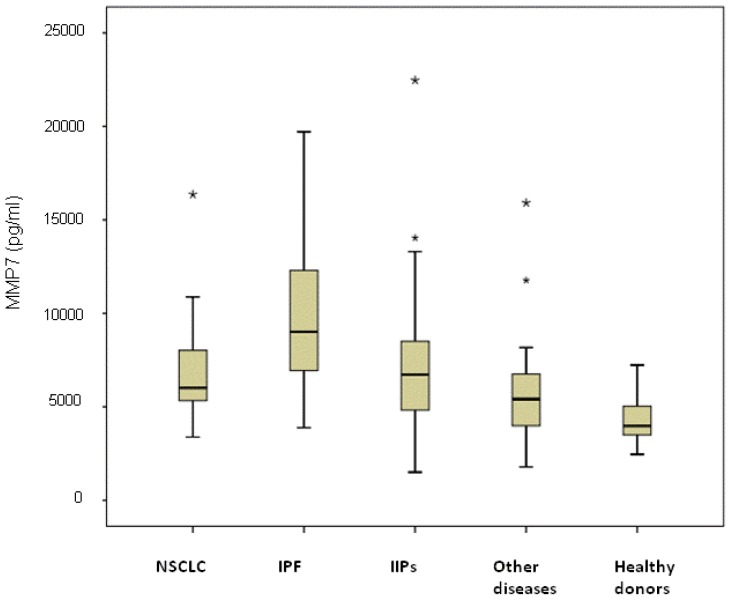
Serum MMP-7 levels in healthy donors and patients with different lung diseases The asterisks stand for outliers, *i.e*. observation points that are distant from other observations.

The highest median value was observed in the subgroup of IPF patients (9100 pg/mL, range 3880–19,720 pg/mL) and was significantly higher than that of NSCLC patients (6310 pg/mL, range 3380–16,360 pg/mL; *p* < 0.0001), IIP patients (6500 pg/mL, range 1500–22,470 pg/mL; *p* = 0.007), individuals with other diseases (5410 pg/mL, range 1780–15,910 pg/mL; *p* < 0.0001) and healthy donors (4350 pg/mL, range 2450–7230 pg/mL; *p* < 0.0001) (Figure 2). Serum MMP-7 levels in NSCLC were significantly higher than those of other diseases and healthy donors (*p* = 0.041 and *p* < 0.0001, respectively) and were similar to those of NSIP patients (*p* = 0.621). Among NSCLC patients, no differences were observed between patients with or without COPD, with median values of 5862 pg/mL (range 3380–10,875 pg/mL) and 6025 pg/mL (range 3790–16,360 pg/mL), respectively (*p* = 0.635). Moreover, considering all COPD patients, no differences in MMP-7 median values were observed in those with cancer (5862 pg/mL (range 3380–10,875 pg/mL)) with respect to patients with COPD alone (5482 pg/mL, range 4544–15,910 pg/mL) (*p* = 0.150).

No correlation was found with the clinical-pathological characteristics of patients or with clinical features of the various diseases (data not shown).

### 2.3. Diagnostic Potential of fcDNA

**Figure 3.**
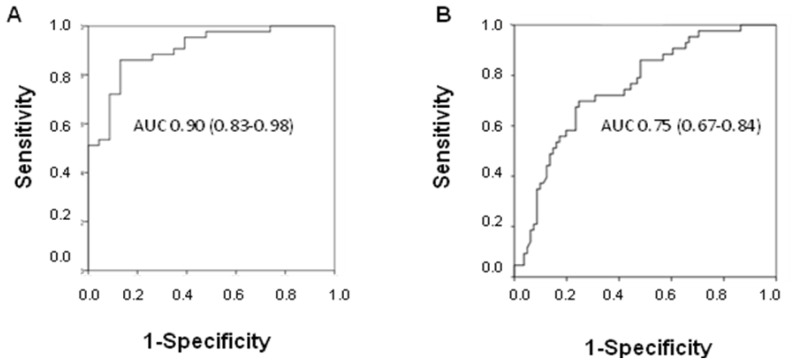
Diagnostic potential of free circulating DNA in discriminating between non-small cell lung cancer patients and (**A**) healthy donors, or (**B**) individuals with other diseases.

The diagnostic power of fcDNA was analyzed by ROC curve analysis. A high area under the curve (AUC) value was observed in discriminating between NSCLC patients and healthy donors (AUC 0.90; 95% CI 0.83–0.98), whereas the value decreased when individuals with other lung diseases were included in the control group (AUC 0.75; 95% CI 0.67–0.84) (Figure 3A,B). Moreover, considering all COPD patients, it was seen that fcDNA discriminated between those with cancer and patients with COPD alone (AUC 0.73; 95% CI 0.53–0.93).

fcDNA efficiently discriminated between IPF patients and healthy donors (AUC 0.87; 95% CI 0.78–0.96), whereas its diagnostic potential in differentiating IPF patients from those with IIPs including NSIPs or other diseases was poorer. In particular, AUC values of 0.65 (95% CI 0.50–0.80) and 0.64 (95% CI 0.51–0.78) were obtained in discriminating IPF patients from those with NSIP or from individuals with other non-neoplastic diseases, respectively (data not shown).

The diagnostic potential of fcDNA was also analyzed in terms of sensitivity and specificity. Good sensitivity and specificity values were obtained in distinguishing NSCLC patients from healthy donors, ranging from 80% to 90% for the best cut off value. Specificity decreased when the different lung diseases were considered as the control group, with a value of around 80% only at the cut off of 100 ng/mL, characterized by 56% sensitivity (Table 2). At the same cut off, fcDNA distinguished IPF patients (56% sensitivity) form healthy donors, IIPs and other diseases (96%, 88% and 77% specificity, respectively) (Table 3).

### 2.4. Diagnostic Potential of Serum MMP-7

**Figure 4.**
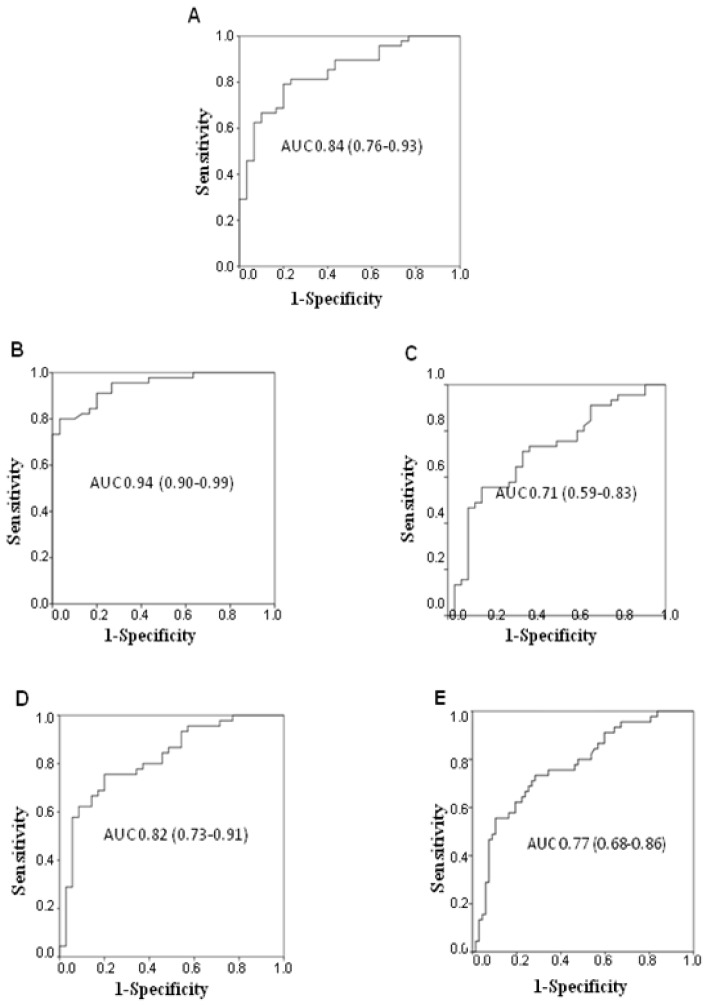
Diagnostic accuracy of serum MMP-7 levels in discriminating between non-small cell lung cancer patients and (**A**) healthy donors, and between idiopathic pulmonary fibrosis patients and (**B**) healthy donors; (**C**) non-specific interstitial pneumonia patients; (**D**) individuals with other non-malignant lung diseases; and (**E**) non-specific interstitial pneumonia patients together with individuals with other non-malignant lung diseases.

The diagnostic potential of MMP-7 was analyzed in terms of its ability to discriminate between the different lung diseases. A high AUC value was observed comparing NSCLC patients and healthy donors (AUC 0.84; 95%, CI 0.76–0.93) (Figure 4A), whereas the diagnostic potential significantly decreased when cancer patients were compared to healthy donors and individuals with NSIP or other lung diseases (AUC 0.64; 95% CI 0.55–0.73).

Higher diagnostic accuracy was observed for IPF patients compared to healthy donors (AUC 0.94; 95% CI 0.90–0.99), NSIP (AUC 0.71; 95% CI 0.59–0.83), other lung diseases (AUC 0.82; 95% CI 0.73–0.91), or NSIP and other lung diseases together (AUC 0.77; 95% CI 0.68–0.86) (Figure 4B–E).

We also analyzed the diagnostic potential of MMP-7 in terms of its sensitivity and specificity in discriminating between NSCLC patients and healthy donors or individuals with other lung disease together with other IIPs including NSIP and chronic HP patients (Table 4). Acceptable sensitivity and specificity values were observed when healthy donors were considered as the control group, whereas very low specificity levels were registered when the control group was composed of the group of the other diseases. Higher values were obtained in discriminating between IPF patients and the other groups. In particular, sensitivity of 56% and 47% was observed at the cut off values of 9000 and 10,000 pg/mL, respectively, with specificity reaching 91% for NSIP and the other diseases, 94% for NSCLC patients and 100% for healthy donors (Table 5).

### 2.5. MMP7 Level and fcDNA Quantity Combination

We also analyzed the relation between MMP-7 serum levels and fcDNA quantity, noting that they were significantly correlated in IPF patients (*r* = 0.428, *p* = 0.014).

The combination of MMP-7 and fcDNA was evaluated for its potential to discriminate between NSCLC patients and those with other lung diseases and healthy donors.

When the markers were used as continuous variables, transformed into natural logarithmic values and analyzed in a multiple logistic regression model, adjusted for age, gender and smoking habits, a unit increase in log DNA and log MMP-7 was associated with a 3.84- and 2.86-fold increase in cancer risk, respectively (Table 6).

ROC curve analysis showed no improvement in accuracy when fcDNA was analyzed in combination with MMP-7 (AUC ROC 0.77; 95% CI 0.69–0.86) with respect to fcDNA alone (*p* = 0.549), whereas the difference became statistically significant compared to MMP-7 alone (*p* = 0.009), confirming that fcDNA on its own is as accurate as the combination of the two markers.

We also analyzed the diagnostic potential of the two markers in combination to discriminate between IPF patients and the other lung diseases. Comparing IPF and NSIP patients, no increase in diagnostic accuracy was seen for the combination analysis (AUC 0.76; 0.63–0.89) with respect to the 2 markers used singly (*p* = 0.776 and *p* = 0.182 for MMP-7 and fcDNA, respectively). Moreover, comparing IPF patients with those with NSIP and other lung diseases, the combined ROC curve (AUC 0.79; 95% CI 0.69–0.90) was similar to that of MMP-7 alone (*p* = 0.876) and significantly higher than that of fcDNA (*p* = 0.019), suggesting that MMP-7 alone is a good discriminating marker for IPF. When the group of healthy donors was added to the analysis, the combined ROC curve was 0.84 (95% CI 0.75–0.92), again similar to that of MMP-7 alone. In the multiple logistic regression model adjusted for age, gender and smoking habits, a unit increase in log DNA and log MMP-7 was associated with a 2.21- and 15.14-fold increase in cancer risk, respectively (Table 7).

## 3. Discussion

In the present study we compared MMP-7 and fcDNA levels in patients with different pulmonary diseases, including IPF, other IIPs including iNSIP and chronic HP and NSCLC, with those of healthy individuals. A diagnosis of IPF currently requires a coordinated multidisciplinary consensus approach and cannot rely solely on the recognition of a UIP pattern found in surgical lung biopsies or on a HRCT scan [18]. Moreover, the histological diagnosis of UIP is severely limited by low sensitivity, specificity and reproducibility, even when performed by experts [19–21]. The identification of a non-invasive and more accurate approach to the differential diagnosis of IPF has thus become an important goal, and several studies have addressed this issue by searching for effective tissue or circulating biomarkers that are specific for this disease [22–24]. Lung cancer is the most frequently diagnosed tumor and the leading cause of cancer mortality throughout the world. The 5-year survival rate is only about 16% for patients diagnosed with advanced lung cancer compared to 70%–90% when the disease is diagnosed and treated at an earlier stage [25]; hence the importance of accurate, easily usable diagnostic tools for early detection.

We previously demonstrated that fcDNA levels are higher in patients with lung cancer than in healthy individuals (including heavy smokers), suggesting that this marker could be an important tool in screening programs to identify lung cancer [9].

In another study we observed significantly higher levels of fcDNA in patients with IPF than in those with other non-specific pulmonary fibrosis diseases such as iNSIP, other diffuse/non-malignant lung diseases, or in healthy controls [7]. We also showed the good diagnostic accuracy of this marker in discriminating between IPF patients and individuals with other lung diseases, including NSIP. The high fcDNA values found in IPF were similar to those found in patients with lung cancer. In the present study, in an independent case series, we confirmed that patients with IPF or early stage NSCLC have similar fcDNA levels, which are also higher than those of individuals with NSIP or other lung diseases and healthy donors. These results indicate that fcDNA may be capable of discriminating between NSCLC and other lung diseases (excluding IPF), or of distinguishing IPF patients from those with NSIP or other benign lung diseases. Furthermore, fcDNA values were higher in COPD patients with lung cancer than in patients with COPD alone, suggesting a role of the marker as a diagnostic tool in this subgroup of patients.

Such findings could be of importance considering the lack of non-invasive biomarkers available for the differential diagnosis between NSCLC and other benign lung disease or between IPF and NSIP.

The expression of metalloproteinases in plasma has been indicated as a potentially important tool for IPF differential diagnosis. Rosas and coworkers reported that increased peripheral blood levels of two markers, MMP-1 and MMP-7, was also found in the lung of IPF patients and would seem to be specific for IPF [11]. In particular, MMP-7 was correlated with disease severity and was higher in patients with subclinical interstitial lung disease (ILD), although it must be pointed out that Rosas’ results were obtained in a case series that did not include NSIP. In our study, serum MMP-7 levels were higher in IPF than in ILD, including NSIP. The marker was also higher in IPF than in NSCLC patients, demonstrating a specificity for the former disease. At the cut off characterized by about 50% sensitivity, >90% specificity was observed in distinguishing IPF from NSIP or other diseases, while absolute specificity was observed with respect to healthy donors.

The combined analysis of the two markers revealed that fcDNA represented the best diagnostic marker for the differential diagnosis between NSCLC and the other lung diseases, while the addition of MMP-7 did not give any further significant information. Conversely, with regard to the differential diagnosis between IPF and NSIP or the other diseases, MMP-7 proved to be the best diagnostic marker, the addition of fcDNA did not improve results. We also observed that fcDNA was only significantly correlated with MMP-7 in IPF patients, suggesting that DNA released in IPF patients may involve processes in which MMP-7 is also involved. It has been shown that pro-MMP-7 is activated in the lung tissue of IPF patients and that MMP-7 contributes to the formation of hyperplastic foci and tissue repair in this disease [15]. Its activity against various ECM components including basement membrane collagen IV, elastin, proteoglycans and other cell adhesion molecules indicates a potential correlation with fcDNA release. Other mechanisms, such as aberrant angiogenesis associated with defective epithelial repair or epithelial proliferation, macrophage activation, fibroblastic accumulation, or an increased level of circulating fibrocytes in the peripheral blood, may also play a role in the release of fcDNA in IPF patients. Conversely, the release of DNA in cancer patients, due mainly to apoptosis and necrosis processes, may explain the lack of correlation with MMP-7, which is minimally involved in these types of processes.

Our results confirmed the role of MMP-7 as a diagnostic marker for IPF and also highlighted the relevance of fcDNA in both NSCLC and IPF diagnosis. fcDNA levels could represent a promising non-invasive diagnostic marker to discriminate between early stage NSCLC and other non-neoplastic pulmonary diseases, and also to distinguish IPF patients from those with other IIPs.

To our knowledge this is the first study in which fcDNA and MMP-7 levels were both measured in patients with IPF, NSCLC and other non-specific pulmonary fibrosis diseases such as iNSIP. Our results suggest that IPF is not simply an inflammatory disorder but rather a complex process that resembles malignant lung disease. In support of this hypothesis, it has also been observed that a number of markers of epithelial instability are overexpressed in lung tissue from patients with IPF/UIP. Moreover, *KRAS* and *TP53* gene mutations have been detected in alveolar type II pneumocytes of lung tissue from IPF patients, and their protein upregulation, which causes an imbalance in different growth factors, has also been associated with increased lung tumorigenesis [24]. Finally, we also demonstrated that abnormal bronchiolar proliferation is a major event in IPF triggered by molecular abnormalities of the wnt/beta-catenin and p53/p21waf1 pathways and involving progenitor basal cells at the bronchioloalveolar junction [26].

## 4. Experimental Section

### 4.1. Case Series

Patients were recruited from the Department of Diseases of the Thorax of Morgagni-Pierantoni Hospital in Forlì, Italy, from January 2008 to January 2012. All procedures relating to informed consent, data collection and privacy protection were approved by the institutional Research Ethics Board. The following cases were analyzed: 48 early stage (IA or IB) NSCLC, 45 IPF, 30 other forms of idiopathic interstitial pneumonia (IIPs) including 24 idiopathic NSIP (iNSIP) and 6 chronic hypersensitivity pneumonitis (HP), and 35 diffuse/non-malignant lung disease (miscellaneous), including 10 pulmonary infection, 13 chronic obstructive pulmonary disease (COPD), 4 asthma, 2 cryptogenetic organizing pneumonia (COP), 4 sarcoidosis and 2 asbestosis. Thirty healthy donors were enrolled during the same period from the Blood Transfusion Unit of the same hospital. Clinical characteristics of patients and healthy donors are shown in Table 1.

### 4.2. NSCLC

Forty-eight resectable stage I (24 IA and 24 IB) NSCLC patients were enrolled onto the study. Histological diagnosis was as follows: adenocarcinoma (36 patients), squamous cell carcinoma (11 patients) and sarcomatoid carcinoma (1 patient).

### 4.3. Interstitial Lung Diseases

A diagnosis of IPF, other IIPs (including iNSIP and chronic HP) and other diffuse lung diseases (COP) was made according to the guidelines of the American Thoracic Society/European Respiratory Society [27,28]. Surgical lung biopsies had been performed cumulatively in 52 patients to establish a diagnosis of usual interstitial pneumonia (UIP) (29/45), iNSIP (22/22) or chronic HP (3/6). In particular, HP showed the following features: (a) positive serum-specific precipitating antibodies; (b) clinical and functional features of diffuse lung disease; (c) High resolution CT scan (HRCT) showing diffuse, poorly defined centrilobular micronodules, ground glass attenuation, focal air trapping, and mild to moderate fibrotic changes; and (d) >30% lymphocytes in bronchoalveolar lavage (BAL) fluid. Three of the six patients had undergone a surgical lung biopsy and in all cases lung histology was consistent with a diagnosis of HP. Diagnosis and staging of sarcoidosis were determined according to American Thoracic Society and European Respiratory Society criteria [29,30]. Diagnosis was histologically confirmed by transbronchial lung biopsy and/or transbronchial needle aspiration via fiber bronchoscope. The sarcoidosis cohort included 4 patients with stage II disease, classified according to the chest radiographic staging system, and 1 patient with stage III disease. HRCT scans, performed within 30 days of peripheral blood sample for DNA extraction, were evaluated by 2 expert radiologists. Surgical lung biopsies were reviewed by an expert pathologist.

At the time of the study, none of the IPF patients were taking immunosuppressive drugs.

### 4.4. Functional Data

The average forced vital capacity (FVC) and carbon monoxide diffusing capacity (DLCO) was 74% ± 16.8% and 60.1% ± 18.3% for IPF, 77% ± 20.4% and 59% ± 14% for iNSIP, 72% ± 18.1% and 54.2% ± 16.3% for chronic HP, and 78.3% ± 18.1% and 74.7% ± 15.7% for sarcoidosis, respectively.

### 4.5. Chronic Obstructive Pulmonary Disease

The diagnosis of COPD was based on the GOLD guidelines [31]. Individuals were clinically stable at the time of examination and there were no clinical or laboratory diagnoses of collagen vascular disease, infections, or other systemic inflammatory diseases. The COPD cohort included 3 patients with GOLD class I, 9 with GOLD II, and 1 with GOLD III–IV.

### 4.6. Asthma

The diagnosis of asthma was based on the GINA guidelines [32]. Individuals were clinically stable at the time of examination (controlled asthma) and there were no clinical or laboratory diagnoses of collagen vascular disease, infections, or other systemic inflammatory diseases.

### 4.7. Pulmonary Infection

The diagnosis of pulmonary infection was based on the presence of febrile pulmonary infiltrates resolved after appropriate antibiotic therapy.

### 4.8. Blood Sample Collection

Five milliliters of peripheral blood were collected from each individual in SST collection tubes. After one hour, when clotting was complete, the tubes were centrifuged at 1500× *g* to obtain serum, which was stored at −80 °C until use.

### 4.9. Serum MMP-7 Assay

The quantitative determination of MMP-7 level in serum was performed using Quantikine Human Total MMP-7 kit (R&D Systems, Minneapolis, MN, USA) according to the manufacturer’s instructions.

### 4.10. fcDNA Analysis

Quantification of fcDNA was performed as previously reported [7,9]. Briefly, DNA was extracted from 1 mL of serum by QIamp DNA Mini Kit (Qiagen, Hilden, Germany) and quantified using a Real-Time quantitative PCR assay based on SYBR Green I dye chemistry (BioRad, Milan, Italy) by amplifying the glyceraldehyde-3-phosphate dehydrogenase (*GAPDH*) gene. The absolute concentration of target DNA was calculated on a standard curve using DNA concentrations ranging from 0.01 to 25 ng obtained from the peripheral blood of a healthy donor. Each sample was run in triplicate and intra-assay variability was assessed by computing the coefficient of variation (CV) among the three *C*_t_ values (defined as the fractional cycle number at which the emitted fluorescence exceeds a fixed threshold value above the baseline), which was always <1.5%. Inter-assay variability between two independent experiments in which the procedure was repeated using another sample from the same individual was assessed by computing the CV, which was always <15%. All measurements were made blind.

### 4.11. Statistical Analysis

Distribution of serum fcDNA and MMP-7 values was evaluated graphically with box plots. Non-parametric ranking statistics (median test) were used to analyze the relationship between median values of fcDNA and serum MMP-7 in healthy donors and patients. Spearman’s correlation coefficient (*r*_s_) was used to investigate the relationship between the two biomarkers considered as continuous variables. The most efficient cut off values to discriminate between the different groups of diseases and healthy donors were determined using receiver operating characteristic (ROC) curve analysis. The true positive rates (sensitivity) were plotted against the false positive rates (1-specificity) for all classification points. 95% confidence intervals (95% CI) were calculated for sensitivity and specificity values.

The independent diagnostic relevance of markers considered as continuous variables was analyzed by the logistic regression model in which natural logarithmic concentrations of markers were considered as predictor variables, and cancer status (case/control) was considered as a binary outcome variable. The linear predictor or logit resulting from this multivariable model after stepwise procedure was used as a new diagnostic test for which the ROC curve was calculated.

All *p* values were based on two-sided testing and statistical analyses were carried out using SAS Statistical software version 9.1 (SAS Institute, Milan, Italy) and the Statistical Package for Social Science (SPSS, version 20.0; Milan, Italy).

## 5. Conclusions

In conclusion, we confirmed the role of fcDNA as a diagnostic marker for NSCLC in a case series composed of early-stage NSCLC. Our results reinforce the potential usefulness of this marker for the early detection of lung cancer and for the evaluation of cases of dubious diagnosis or of suspicious nodules detected by spiral CT. fcDNA levels could also play a role in discriminating between IPF and other IIPs, including NSIP and chronic HP. MMP-7 serum levels do not further increase the diagnostic potential of fcDNA in NSCLC, but would seem to play an important role in the differential diagnosis between IPF and other types of ILD. Considering the clinical importance of a differential diagnosis between IPF and other IIPs, our findings highlight the possibility of having a non-invasive diagnostic test that can aid diagnosis. These biomarkers also have the potential to greatly facilitate the introduction of new therapies and to profoundly affect the management of patients with IPF or other IIPs.

## Figures and Tables

**Table 1.** Clinical characteristics of patients and healthy donors.

Clinical Characteristics	NSCLC *N*	IPF *N*	IIPs *N*	Other Diseases *N*	Healthy Donors *N*
Gender					
F	21	5	15	14	13
M	27	40	15	21	17
Age, years					
>65	31	22	14	13	12
≤65	17	23	16	22	18
Smoking habits					
Never	10	12	14	10	11
Former	25	31	15	15	13
Current	13	2	1	10	6
Total	48	45	30	35	30

NSCLC, non small cell lung cancer; IPF, idiopathic pulmonary fibrosis; IIP, idiopathic interstitial pneumonia.

**Table 2.** Sensitivity and specificity values of free circulating DNA in discriminating between NSCLC patients (43) and healthy donors or individuals with other lung diseases.

Cut Off (ng/mL)	Sensitivity %	Specificity %	

		Healthy Donors (*n* = 23)	Other Diseases Including NSIP (*n* = 58)
60	79	87	40
70	74	91	52
80	70	91	69
90	58	91	76
100	56	96	81

**Table 3.** Sensitivity and specificity values of free circulating DNA in discriminating between IPF patients (32) and healthy donors, or individuals with other non-malignant lung diseases.

Cut Off (ng/mL)	Sensitivity %	Specificity %			

		Healthy Donors (*n* = 23)	NSIP (*n* = 24)	Other Diseases (*n* = 34)	Other Diseases Including NSIP (*n* = 58)
80	56	91	75	65	69
90	56	91	83	71	76
100	56	96	88	77	81
110	47	100	88	77	81

**Table 4.** Sensitivity and specificity values of serum MMP-7 levels in discriminating between NSCLC patients (48) and healthy donors or individuals with other lung diseases.

Cut Off (pg/mL)	Sensitivity %	Specificity %	

		Healthy Donors (*n* = 30)	Other Diseases Including NSIP (*n* = 65)
4000	94	53	19
5000	85	73	40
6000	54	93	51

**Table 5.** Sensitivity and specificity values of serum MMP-7 levels in discriminating between IPF patients (45) and healthy donors or individuals with other non-malignant lung diseases.

Cut Off (pg/mL)	Sensitivity %	Specificity %				

		Healthy Donors (*n* = 30)	NSIP (*n* = 30)	Other Diseases (*n* = 35)	Other Diseases Including NSIP (*n* = 65)	NSCLC (*n* = 48)
8000	67	100	67	83	76	75
9000	56	100	84	91	88	85
10,000	47	100	91	91	91	94
11,000	36	100	91	91	91	98

**Table 6.** Combined analysis of MMP-7 and free circulating DNA, considered as continuous variables in a multiple logistic regression model adjusted for age, gender and smoking habits of NSCLC patients compared to the other lung diseases (except IPF) and healthy donors.

Variable	Coefficient (Standard Error)	Wald Test	OR (95% CI)	*p*
Constant	−15.48137	−3.05	-	-
ln MMP7	1.04992	1.87	2.86 (0.95–8.60)	0.062
ln DNA	1.344679	4.09	3.84 (2.01–7.30)	<0.0001

LOGIT = −15.48137 + 1.04992 ln MMP-7 + 1.344679 ln DNA.

**Table 7.** Combined analysis of MMP-7 and free circulating DNA, considered as continuous variables in a multiple logistic regression model adjusted for age, gender and smoking habits of IPF patients compared to the other lung diseases and healthy donors.

Variable	Coefficient (Standard Error)	Wald Test	OR (95% CI)	*p*
Constant	−28.23784	−4.63	-	-
ln MMP7	2.717137	3.97	15.14 (3.95–57.94)	<0.0001
ln DNA	0.7944188	2.16	2.21 (1.07–4.56)	0.031

LOGIT = −28.23784 + 2.717137 ln MMP-7 + 0.7944188 ln DNA.
